# Heterogeneous multiple kernel learning for breast cancer outcome evaluation

**DOI:** 10.1186/s12859-020-3483-0

**Published:** 2020-04-23

**Authors:** Xingheng Yu, Xinqi Gong, Hao Jiang

**Affiliations:** 1 Mathematics Intelligence Application Lab, Institute for Mathematical Sciences, Renmin University of China, No.59 ZhongGuanCun Avenue, HaiDian District, Beijing, 100872 China; 2 School of Mathematics, Renmin University of China, No.59 ZhongGuanCun Avenue, HaiDian District, Beijing, 100872 China

**Keywords:** HMKL, MKL, PSO, Hadamard kernel, Breast Cancer

## Abstract

**Background:**

Breast cancer is one of the common kinds of cancer among women, and it ranks second among all cancers in terms of incidence, after lung cancer. Therefore, it is of great necessity to study the detection methods of breast cancer. Recent research has focused on using gene expression data to predict outcomes, and kernel methods have received a lot of attention regarding the cancer outcome evaluation. However, selecting the appropriate kernels and their parameters still needs further investigation.

**Results:**

We utilized heterogeneous kernels from a specific kernel set including the Hadamard, RBF and linear kernels. The mixed coefficients of the heterogeneous kernel were computed by solving the standard convex quadratic programming problem of the quadratic constraints. The algorithm is named the heterogeneous multiple kernel learning (HMKL). Using the particle swarm optimization (PSO) in HMKL, we selected the kernel parameters, then we employed HMKL to perform the breast cancer outcome evaluation. By testing real-world microarray datasets, the HMKL method outperforms the methods of the random forest, decision tree, GA with Rotation Forest, BFA + RF, SVM and MKL.

**Conclusions:**

On one hand, HMKL is effective for the breast cancer evaluation and can be utilized by physicians to better understand the patient’s condition. On the other hand, HMKL can choose the function and parameters of the kernel. At the same time, this study proves that the Hadamard kernel is effective in HMKL. We hope that HMKL could be applied as a new method to more actual problems.

## Background

An estimated number of 246,660 patients will be diagnosed with breast cancer in the United States each year, with > 40,000 estimated cancer-related deaths [1]. Early detection and identification of breast cancer are essential to reduce the consequences of the disease. On the other hand, the prognosis of cancer can help to design the treatment programs, which is also very important. Cancer prognosis can be explained as estimating the probability of survival among the patients over a period of time after surgery. The DNA microarray technology for the breast cancer diagnosis has turned into a very prevalent research topic, as it simultaneously measures the expression of a lot of genes and leads to a high-quality cancer identification. However, the number of genes ranges from 1000 to 10,000, while the number of samples is often less than 200.

A lot of effort has been made on the analysis based on gene expression profiling [2–7] to predict the prognosis of breast cancer patients. Broët et al. [8] tried to identify the gene expression features in a microarray dataset, Jagga et al. [9] exploited correlation-based algorithms, and Bhalla et al. [10] exploited threshold-based algorithms to predict the prognosis of breast cancer patients.

Multiple kernel learning (MKL) algorithms have been proved to be effective tools to solve learning problems such as classification or regression. Jérôme Mariette et al. [11] applied MKL on breast cancer heterogeneous data and achieved a good performance through the experiments. Arezou et al. [12] proposed an MKL method, which employs the gene expression profiles to predict cancer and achieves a satisfactory predictive performance. Their MKL gene set algorithm was compared with the two standard algorithms of random forest and SVM for the cancer genome Atlas queues. On average, MKL can achieve a higher evaluation performance than other methods. Therefore, in this work we consider using MKL as the control group of our algorithm (HMKL). In MKL, it is essential to select the set of kernel functions and optimize the mixed coefficients. Rakotomamonjy et al. [13] proposed an efficient algorithm called SimpleMKL, which utilizes the gradient descent of the SVM target value, to be applied to the MKL problem. Using the reduced gradient descent, the mixed coefficient of the kernels in the standard SVM solver was iteratively determined. They employed the applied alternative optimization algorithm to optimize the parameters, and this could be applied to the Multiple Kernel Learning Primal Problem using the reduced gradient algorithm. It also shows that the generalization performance of this method is similar to or better than that obtained by cross-validation when the parameters of the heterogeneous kernel are selected.

In the current view, the effectiveness of the kernel methods depends on the choice of the kernel. Jiang et al. [14] proposed the Hadamard Kernel SVM to predict the prognosis of breast cancer patients based on the gene expression profiles. The Hadamard Kernel is better than the classical kernels considering the ROC curve (AUC), but determining the optimal parameters of the kernels needs further discussions. Besides, it is usually accepted that single kernels describe only one side information of the data. When the kernels are integrated, the performance may be improved by providing a better description of the nonlinear and complex data relationships. Kennedy et al. [15] discovered the particle swarm optimization (PSO) through the simulation of a simplified social model. Lin et al. [16] utilized PSO to increase the classification accuracy rate in SVM, in a method called PSO + SVM. The developed PSO + SVM can adjust the kernel function parameters; thus, PSO can be applied to select the kernel parameters.

Emina et al. [17] used the GA feature selection and Rotation Forest to diagnose breast cancer. They have proposed several data mining methods with and without GA-based feature selection to correctly classify the medical data (the data was taken from the Wisconsin Diagnostic Breast Cancer database). The random forest and GA feature selection gave the highest accuracy. Sawhney et al. [18] explored the inclusion of a penalty function to the existing fitness function promoting the Binary Firefly Algorithm to drastically reduce the feature set to an optimal subset, and their results showed an increase in both classification accuracy and feature reduction using a random forest classifier for the diagnosis of breast, cervical and hepatocellular carcinoma.

In this paper, we build a new model named HMKL, which employs three heterogeneous kernels including the Hadamard Kernel, RBF and linear kernels to improve the AUC of the evaluation. Additionally, we employ PSO to solve the problem of selecting the kernel parameters. The remainder of the paper is organized as follows. In the “Methods” section, we explain the mathematical model and the calculation process of HMKL. In the “Results” section, we demonstrate the performance of the evaluation through common datasets.

## Methods

**Fig. 1:**
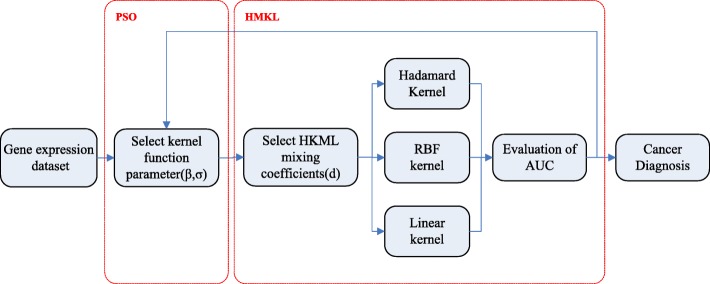
The general schema of HMKL. The HMKL framework consists of two parts. The first part is to select the optimal kernel function parameters by PSO and the second part is an HMKL framework composed of three heterogeneous kernels (Hadamard, RBF and linear kernels)

In this section, we introduce a new algorithm for integrating multiple kernels, which we call HMKL. This method combines three kernels that are the Hadamard, RBF and linear kernels, and it is capable of learning the best kernel by optimizing the kernel parameters and weight parameters embedded in the kernel set, providing a better description of the nonlinear relationship among the gene expression data. Figure 1 shows the general schema of our algorithm HKML.

We utilize an optimization algorithm to calculate the HMKL framework in two steps and obtain the best parameters of the kernels. In order to determine the parameters of the kernel function, we employ the PSO algorithm in HMKL.

The kernel matrix is constructed based on the measure of pairwise relationship. Different types of kernels reflect different kinds of data relationships. The linear kernel measures the linear correlation in the data, and when the dataset is not linearly separable, the non-linear mapping of the input vectors can be constructed into a feature space of a higher dimensionality.

The kernels utilized in HMKL include:

Hadamard kernel:

$$ {K}_1\left({\mathrm{x}}_i,{\mathrm{x}}_j\right)={K}_{\beta}\left({\mathrm{x}}_i,{\mathrm{x}}_j\right)=\sum \limits_{k=1}^p\frac{\left|{x}_{ik}\right.\left|{}^{\beta}\right.\left|{x}_{ik}\right.\left|{}^{\beta}\right.}{2\left(\left|{x}_{ik}\right.\left|{}^{\beta}\right.+{\left|x\right.}_{ik}\left|{}^{\beta}\right.\right)},i,j=1,2,\cdots, N $$

RBF kernel:

$$ {K}_2\left({\mathrm{x}}_i,{\mathrm{x}}_j\right)={K}_{\sigma}\left({\mathrm{x}}_i,{\mathrm{x}}_j\right)=\exp \left(\frac{\left\Vert {x}_i-{x}_j\right\Vert }{2{\sigma}^2}\right) $$

Linear kernel:

$$ {K}_3\left({\mathrm{x}}_i,{\mathrm{x}}_j\right)=K^{\prime}\left({\mathrm{x}}_i,{\mathrm{x}}_j\right)={x_i}^{\mathrm{T}}{x}_j $$

We employ the above-mentioned three kernel functions in the HMKL to obtain the combined kernel which can describe both the linear and nonlinear relationships in the data. The two kernel parameters (β, *σ*) in the kernel set need to be predefined before MKL, and we employ PSO to select them.

In the PSO algorithm, each particle is represented by its coordinates in a 2-dimensional space. The status of each particle is characterized in accordance with its position and velocity. t represents the current genetic algebra, and we set the maximum number of genetic algebras to MAXGEN. i represents the number of particles. The parameter ${\beta}_i^t$ represents the value of the Hadamard kernel parameter β for the particle i at iteration t. ${\sigma}_i^t$ represents the value of the RBF kernel parameter σ for the particle i at iteration t. ${Z}_i^t=\left\{{\beta}_i^t,{\sigma}_i^t\right\}$ represents the space position for the particle i at iteration t. ${v}_i^t=\left\{{v}_{i\beta}^t,{v}_{i\sigma}^t\right\}$ represents the velocity for the particle i at iteration t. ${v}_{i\beta}^t$ is the optimum value of the Hadamard kernel parameter β changes for the particle i at iteration t. ${v}_{i\sigma}^t$ is the value of the RBF kernel parameter σ changes for the particle i at iteration t. ${P}_i^t=\left\{{P}_{i\beta}^t,{P}_{i\sigma}^t\right\}$ represents the best solution for the particle i at iteration t. ${P}_{i\beta}^t$ represents the value of the Hadamard kernel parameter β changes for the particle i at iteration t. ${P}_{i\sigma}^t$ represents the value of the RBF kernel parameter *σ* changes for the particle i at iteration t. ${P}_g^t=\left\{{P}_{g\beta}^t,{P}_{g\sigma}^t\right\}$ represents the best solution obtained in the population for the particle i at iteration t. ${P}_{g\beta}^t$ represents the optimum value of the Hadamard kernel parameter *β* for all the particles at iteration t of the population. ${P}_{g\sigma}^t$ represents the optimum value of the RBF kernel parameter *σ* for all the particles at iteration t of the population. The velocity of each particle evolves based on the following equations:

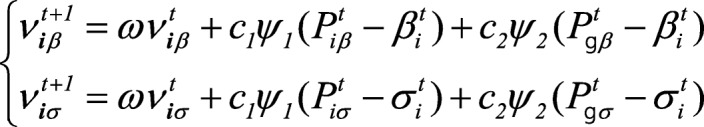



where *c*_1_ represents the cognition learning factor, *c*_2_ represents the social learning factor, *ω* is the inertia weight and *ψ*_1_ and *ψ*_2_ represent random numbers. Each particle then moves to a new potential solution based on the following equations:

$$ \left\{\begin{array}{c}{\beta}_i^{t+1}={\beta}_i^t+{\nu}_{i\beta}^{t+1}\\ {}{\sigma}_i^{t+1}={\sigma}_i^t+{\nu}_{i\sigma}^{t+1}\end{array}\right. $$

### HMKL framework

Let $\mathcal{X}\in {\mathbb{R}}^K$. *ℝ*^*K*^ is the Hilbert space that decomposes into three blocks: ${\mathbb{R}}^K={\mathbb{R}}^{K_1}\times {\mathbb{R}}^{K_2}\times {\mathbb{R}}^{K_3}$. *x* = (*x*_1·_, *x*_2·_, …, *x*_*N*·_) . x_*i*·_ = (*x*_1*i*_, *x*_2*i*_, *x*_3*i*_) such that each *x*_*mi*_, *m* = 1, 2, 3 is a vector. We want to find a linear classifier of the form y = sign(*w*^⊺^*x* + *b*) where $\mathrm{w}=\left({w}_1,{w}_2,{w}_3\right)\in {\mathbb{R}}^{K_1+{K}_2+{K}_3}$. Let ${K}_{\beta_i^t}={K}_1,{K}_{\sigma_i^t}={K}_2,{K}^{\prime }={K}_3$, ${K}_{\beta_i^t},{K}_{\sigma_i^t}$ and *K*^′^ are 3 positive definite kernels.

The data points x_*i*_ are embeddings in a Euclidean space via a mapping $\phi :\mathcal{X}\to {\mathbb{R}}^K$, we assume that $\phi (x)=\left({d}_1^{1/2}\ {\phi}_1(x),{d}_2^{1/2}\ {\phi}_2(x),{d}_3^{1/2}\ {\phi}_3(x)\right)$. The following is the decomposition process of the kernel function:

$$ {\displaystyle \begin{array}{c}K\left({\mathrm{x}}_i,{\mathrm{x}}_j\right)=\sum \limits_{m=1}^3{d}_m{\phi}_m{\left({x}_i\right)}^T{\phi}_m\left({x}_j\right)\\ {}=\sum \limits_{m=1}^3{d}_m{K}_m\left({x}_i,{x}_j\right)\\ {}={d}_1{K}_{\beta_i^{\mathrm{t}}}\left({x}_i,{x}_j\right)+{d}_2{K}_{\sigma_i^{\mathrm{t}}}\left({x}_i,{x}_j\right)+{d}_3K\hbox{'}\left({x}_i,{x}_j\right)\Big)\end{array}} $$

The mixed coefficient *d*_*m*_ ≥ 0, $\sum \limits_{m=1}^3{d}_m=1$*.* Inspired by the framework of Wahba et al. [19] and Rakotomamonjy et al. [13], we propose to solve the following convex problem to address the HMKL problem:

1 $$ {\displaystyle \begin{array}{l}\underset{b,\xi, d,w}{\min}\sum \limits_{m=1}^3\frac{1}{2}{d}_m{\left\Vert {w}_m\right\Vert}^2+c\sum \limits_{i=1}^N{\xi}_i\\ {}s.t.\kern1em w\in {\mathbf{\mathbb{R}}}^{K_{\beta_i^{\mathrm{t}}}+{K}_{\sigma_i^{\mathrm{t}}}+K\hbox{'}},\xi \in {\mathbf{\mathbb{R}}}_{+}^{\mathrm{n}},b\in \mathbf{\mathbb{R}}\\ {}{y}_i\left(\sum \limits_{m=1}^3{w}_m^T{x}_{mi}+b\right)\ge 1-{\xi}_i,\forall i\in \left\{1,\cdots, N\right\}\\ {}{d}_m\ge 0,\kern0.5em \sum \limits_{\mathrm{m}=1}^3{d}_m=1\end{array}} $$

When *d*_*m*_ = 0, ‖w_m_‖^2^ has to be equal to zero. We hope that the vector *d* is a sparsity constraint that will force some values of *d*_*m*_ to be zero, thus encouraging sparse kernel expansions and optimizing the choice of the kernel.

To derive the optimality conditions, we rearrange the problem to yield an equivalent formulation:

2 $$ {\displaystyle \begin{array}{l}\underset{b,\xi, d,w}{\min}\frac{1}{2}{\left(\sum \limits_{m=1}^3{d}_m\left\Vert {w}_m\right\Vert \right)}^2+c\sum \limits_{i=1}^N{\xi}_i\\ {}s.t.\kern1em w\in {\mathbf{\mathbb{R}}}^{K_{\beta_i^{\mathrm{t}}}+{K}_{\sigma_i^{\mathrm{t}}}+K\hbox{'}},\xi \in {\mathbf{\mathbb{R}}}_{+}^{\mathrm{n}},b\in \mathbf{\mathbb{R}}\\ {}{y}_i\left(\sum \limits_{m=1}^3{w}_m^T{x}_{mi}+b\right)\ge 1-{\xi}_i,\forall i\in \left\{1,\cdots, N\right\}\Big)\\ {}{d}_m\ge 0,\kern0.5em \sum \limits_{\mathrm{m}=1}^3{d}_m=1\end{array}} $$

***Theorem****Formulation (2) is equivalent to formulation (1).*

***Proof:***

*By the Cauchy -Schwartz inequality, we know:*

$$ {\displaystyle \begin{array}{c}{\left(\sum \limits_{m=1}^3{d}_m\left\Vert {w}_m\right\Vert \right)}^2={\left(\sum \limits_{m=1}^3{d}_m^{1/2}{\left\Vert {w}_m\right\Vert}^2{d}_m^{1/2}\right)}^2\\ {}\le \left(\sum \limits_{m=1}^3{d}_m{\left\Vert {w}_m\right\Vert}^2\right)\left(\sum \limits_{m=1}^3{d}_m\right)\\ {}\le \sum \limits_{m=1}^3{d}_m{\left\Vert {w}_m\right\Vert}^2\end{array}} $$

${d}_m^{1/2}$*is proportional to* $\left\Vert {w}_m\right\Vert {d}_m^{1/2}$*, that is:*

$$ {d}_m=\frac{\sum \limits_{j=1}^3\left\Vert {w}_j\right\Vert }{\left\Vert {w}_m\right\Vert } $$

which leads to the following function:

$$ \underset{d_m\ge 0,\sum \limits_{m=1}^3{d}_m=1}{\min}\sum \limits_{m=1}^3{d}_m{\left\Vert {w}_m\right\Vert}^2={\left(\sum \limits_{m=1}^3{d}_m\left\Vert {w}_m\right\Vert \right)}^2 $$

*This completes the proof.*

Formulation (2) shows that the mixed-norm penalization of $\sum \limits_{m=1}^3{d}_m\left\Vert {w}_m\right\Vert$ is a soft-thresholding penalizer that leads to a sparse solution, for which the algorithm performs the kernel selection. The formulations (1) and (2) are equivalent; thus, formulation (1) also leads to a sparse solution. This problem can be solved more efficiently.

Formulation (1) is about a dual problem. The dual problem is a key point to derive algorithms and study their convergence properties. Since our formulation (1) is equivalent to the one in the work of Bach et al. [18], they lead to the same dual problem. The Lagrangian of formulation (1) is as follows:

$$ L=\sum \limits_{m=1}^3{d}_m{\left\Vert {w}_m\right\Vert}^2+c\sum \limits_{i=1}^N{\xi}_i+\sum \limits_{i=1}^N{\alpha}_i\left(1-{\xi}_i-{y}_i\sum \limits_{m=1}^3{w}_m^T{x}_{mi}-{y}_ib\right)-\sum \limits_{i=1}^N{v}_i{\xi}_i+\lambda \left(\sum \limits_{m=1}^3{d}_m-1\right)-\sum \limits_{m=1}^3{\eta}_m{d}_m $$

the Lagrangian gives the following dual problem:

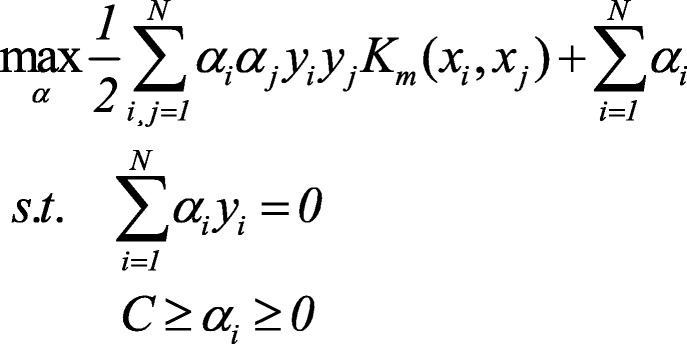



This dual problem is difficult to optimize due to the last constraint, which may be moved to the objective function, but the latter then becomes non-differentiable causing new difficulties [18].

### Algorithm for solving the HMKL problem

Scaling is a usual preprocessing step with important outcomes in many classification methods. Adaptive scaling consists of letting the parameters *d*_*m*_ be adapted during the estimation process with the explicit aim of achieving a better recognition rate. For the HMKL algorithm, *d*_*m*_ is a set of hyperparameters of the learning process. According to the structural risk minimization principle, *d*_*m*_ can be tuned in two ways:

3 $$ \underset{d}{\min }f(d)\kern0.75em such\ that\kern0.5em {d}_m\ge 0,\sum \limits_{m=1}^3{d}_m=1 $$

where

4 $$ f(d)=\left\{\begin{array}{l}\ \underset{b,\xi, w}{\min}\sum \limits_{m=1}^3\frac{1}{2}{d}_m{\left\Vert {w}_m\right\Vert}^2+c\sum \limits_{i=1}^N{\xi}_i\\ {}\ s.t.\kern1em w\in {\mathbf{\mathbb{R}}}^{K_{\beta_i^{\mathrm{t}}}+{K}_{\sigma_i^{\mathrm{t}}}+K\hbox{'}},\xi \in {\mathbf{\mathbb{R}}}_{+}^{\mathrm{n}},b\in \mathbf{\mathbb{R}}\\ {}\kern2.5em {y}_i\left(\sum \limits_{m=1}^3{w}_m^T{x}_{mi}+b\ge 1-{\xi}_i\right),\forall i\in \left\{1,\cdots, N\right\}\end{array}\right. $$

One feasible way to solve the problem (1) is to utilize the quadratic programming of quadratic constraints instead of the optimization algorithm. The first step is to fix *d* and optimize *b*, *ξ* and *w* of problem (1), which can be selected by the SVM parameter optimization algorithms, while the second step is to fix *b*, *ξ* and *w* and optimize d = (*d*_1_, *d*_2_, *d*_3_) to minimize the value of the objective function (4). In the following, we mainly focus on the second step.

In the second step, we note that the Lagrangian of problem (4) is as follows:

$$ L=\sum \limits_{m=1}^3{d}_m{\left\Vert {w}_m\right\Vert}^2+c\sum \limits_{i=1}^N{\xi}_i+\sum \limits_{i=1}^N{\alpha}_i\left(1-{\xi}_i-{y}_i\sum \limits_{m=1}^3{w}_m^T{x}_{mi}-{y}_ib\right) $$

The associated dual problem can then be derived as follows:

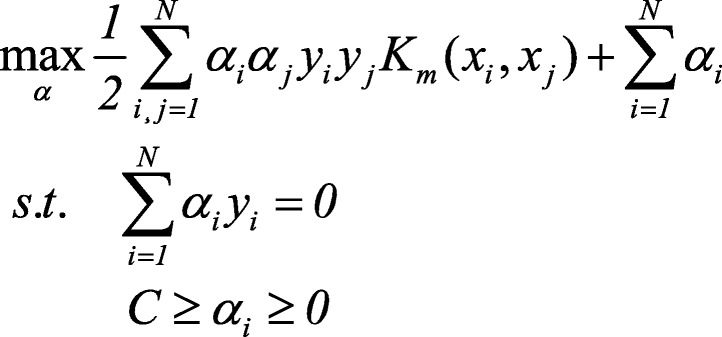



Due to strong duality, *f*(*d*) is the objective value of the dual problem:

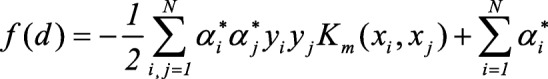



where ${\alpha}_i^{\star }$ maximizes (5), and its derivatives:

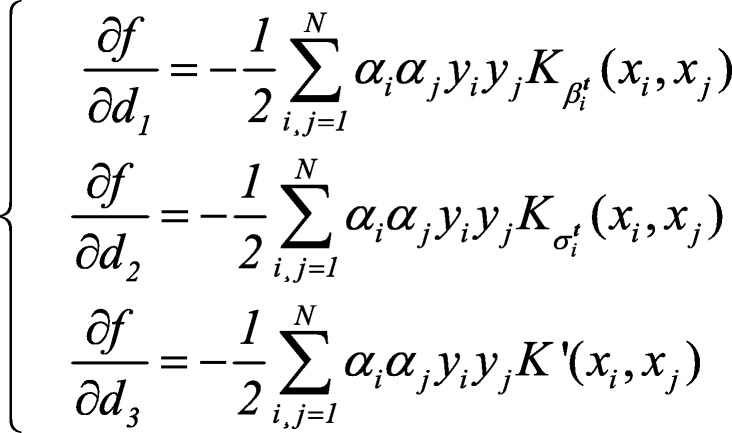



The optimization problem that we have to deal with in (5) is a non-linear objective function with constraints over the simplex. With our positivity assumption on the kernel matrices, *f*(*d*) is convex and differentiable with Lipschitz gradient. The approach we use to solve this problem is a reduced gradient method, which converges for such functions. We employ the method of Bach et al. [20] to update the gradient using the gradient descent algorithm. *d*_*μ*_ represents a non-zero entry of *d*, which is the reduction gradient of *f*(*d*). The components of ∇_*red*_*f* are as follows:

$$ {\left[{\nabla}_{red}f\right]}_m=\frac{\partial f}{\partial {d}_m}-\frac{\partial f}{\partial {d}_{\mu }}\kern0.5em m\ne \mu $$

and

$$ {\left[{\nabla}_{red}f\right]}_{\mu }=\frac{\partial f}{\partial {d}_{\mu }}-\frac{\partial f}{\partial {d}_m}\kern0.5em $$

−∇_*red*_*J* is a descent orientation. The descent orientation for updating d is as follows:

$$ {D}_m=\left\{\begin{array}{c}\ 0\kern1.25em if\ {d}_m=0\ \mathrm{and}\kern0.5em \frac{\partial f}{\partial {d}_m}-\frac{\partial f}{\partial {d}_{\mu }}>0\kern0.5em \\ {}-\frac{\partial f}{\partial {d}_{\mu }}+\frac{\partial f}{\partial {d}_m}\kern0.75em \mathrm{if}\ {d}_m>0\ \mathrm{and}\ \mathrm{m}\ne \mu \\ {}\kern0.5em \sum \limits_{g\ne \mu, {d}_{\mu }>0}\left(\frac{\partial f}{\partial {d}_{\mathrm{v}}}-\frac{\partial f}{\partial {d}_{\mu }}\right)\kern0.75em \mathrm{for}\ \mathrm{m}=\mu \kern1.75em \end{array}\right. $$

The usual updating scheme is d ⟵ d + γD, where γ is the step size. The algorithm is terminated when a stopping criterion is met, which can be either based on the duality gap or the KKT conditions.

### Optimality conditions

**Fig. 2:**
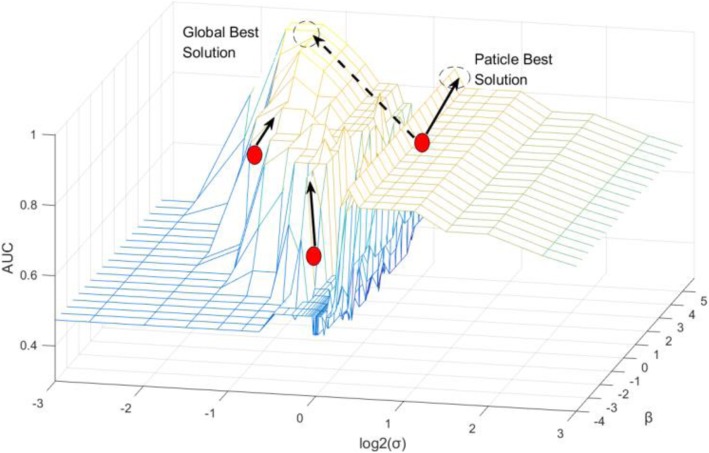
The search concept of the particle swarm optimization. The figure shows how we employ PSO to draw the actual particle selection process of the GSE32394 dataset. There are three particles in each group, and the optimum particle in each group is found in each cycle (Particle Best Solution) and in all the previous cycles of the optimal particle (Global Best Solution)

The proper optimality conditions, such as the KKT conditions or the duality gap, should be zero at the optimum. When deriving the optimality conditions, we rearrange the problem to yield an equivalent formulation. Figure 2 shows the search concept of the particle swarm optimization.

As we note that the Lagrangian of problem (3) is as follows:

$$ L=\sum \limits_{m=1}^3{d}_m{\left\Vert {w}_m\right\Vert}^2+c\sum \limits_{i=1}^N{\xi}_i+\sum \limits_{i=1}^N{\alpha}_i\left(1-{\xi}_i-{y}_i\sum \limits_{m=1}^3{w}_m^T{x}_{mi}-{y}_ib\right)-\sum \limits_{i=1}^N{v}_i{\xi}_i+\lambda \left(\sum \limits_{m=1}^3{d}_m-1\right)=\sum \limits_{m=1}^3{\eta}_m{d}_m $$

The KKT (Karush-Kuhn-Tucker) optimality conditions are therefore as follows:

$$ \left\{\begin{array}{c}\kern0.5em (a)\kern0.75em {d}_m{w}_m=\sum \limits_{i=1}^N{\alpha}_i{y}_i{x}_{mi}\kern1em \\ {}\kern0.5em (b)\kern0.75em \sum \limits_{i=1}^N{\alpha}_i{y}_i=0\kern3.75em \\ {}\kern0.5em (c)\kern0.75em C-{\alpha}_i-{v}_i=0\kern2.5em \\ {}\kern0.5em (d)\kern0.75em \frac{1}{2}{\left\Vert {w}_m\right\Vert}^2+\lambda -{\eta}_m=0\end{array}\right. $$

Known by (a)

$$ \left\{\begin{array}{c}\ \left(\mathrm{A}\right)\kern0.5em {d}_1{w}_1=\sum \limits_{i=1}^N{\alpha}_i{y}_i{K}_{\beta_i^{\mathrm{t}}}\left({x}_i,\cdot \right)\\ {}\ \left(\mathrm{B}\right)\kern0.5em {d}_2{w}_2=\sum \limits_{i=1}^N{\alpha}_i{y}_i{K}_{\sigma_i^{\mathrm{t}}}\left({x}_i,\cdot \right)\\ {}\ \left(\mathrm{C}\right)\kern0.5em {d}_3{w}_3=\sum \limits_{i=1}^N{\alpha}_i{y}_iK\hbox{'}\left({x}_i,\cdot \right)\ \end{array}\right. $$

Whose dual problem is as follows:

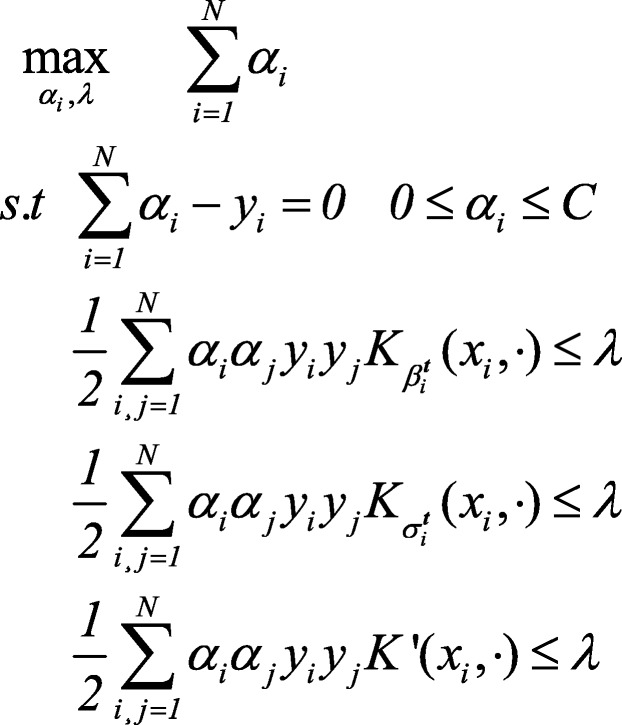



Apart from that, we derive the duality gap in (6) and (7) as follows:

$$ DualGap=f\left({d}^{\ast}\right)-\sum \limits_{i=1}^N{\alpha}_i^{\ast }+\frac{1}{2}\underset{\mathrm{m}}{\max}\sum \limits_{i,j=1}^N{\alpha}_i^{\ast }{\alpha}_j^{\ast }{y}_i{y}_j{K}_m\left({x}_i,{x}_j\right) $$

When the KKT condition and duality gap are satisfied, the optimal solution d = (*d*_1_, *d*_2_, *d*_3_) is obtained.

## Results

### Materials

We retrieved a lot of microarray datasets from The Cancer Genome Atlas (TCGA) and National Center for Biotechnology Information (NCBI) [21]. Table 1 illustrates that the 8 microarray datasets whose accession numbers are GSE32394, GSE1872, GSE59993, GSE76260, GSE59246, BRCA1, BRCA2 and BRCA3 were utilized in the model evaluations. The GSE datasets were obtained from NCBI. In order to test the HMKL algorithm in the NGS datasets, the data were retrieved from TCGA, containing breast cancer samples in various stages, such that each sample was represented by the methylation levels at different CpG sites. We divided the data that were downloaded from TCGA into 3 different test datasets.

**Table 1:** Information about the gene expression datasets

name	Number of genes	Number of samples	Number of classes
GSE32394	1259	19	2
GSE59993	1205	78	2
GSE1872	15,923	35	2
GSE76260	1145	64	2
GSE59246	62,976	102	2
BRCA1	17,204	107	2
BRCA2	17,190	138	2
BRCA3	17,193	223	2

The first dataset GSE32394 is employed to differentiate between the estrogen-receptor-positive (ER+) and estrogen-receptor-negative (ER-) primary breast carcinoma tumors. We can compare two different types of breast cancer using the Custom Affymetrix Glyco v4 array. This dataset has 19 samples.

The second dataset GSE1872 is from an N-methyl-N-nitrosourea-induced breast cancer model, which is utilized to analyze the N-methyl-N-nitrosourea (NMU)- induced primary breast cancer from Wistar-Furth rats females. The number of attributes is 15,923, and there are 35 samples in this dataset.

The third dataset GSE59993 contains circulating miRNA microarray data from breast cancer patients. Independent studies have reported that circulating miRNAs have the potential to be biomarkers. This dataset includes 78 samples (26 hemolyzed and 52 non hemolyzed).

The fourth dataset GSE76260 contains miRNA expression profiling in cancer and non-neoplastic tissues. Summary miRNA expression profiles were evaluated in a series of 64 prostate clinical specimens, including 32 cancer and 32 non-neoplastic tissues.

The fifth dataset GSE59246 is used to differentiate between invasive and non-invasive breast cancer, such that the access number is GSE59246. The mRNA, miRNA and DNA copy number profiles are generated to measure the expression of different samples. The arrays consist of 3 normal controls, 46 ductal carcinoma in situ (CIS) lesions and 56 small invasive breast cancers. We discard the 3 normal controls, so the total number of samples is 102. In this dataset, the number of attributes is 62,976.

The Sixth dataset is BRCA1, which contains the comparison between normal samples and samples at stage VI in terms of BRCA1. This dataset involves 107 samples in total from TCGA, among which 11 are stage VI and 96 are normal samples. and the number of genes is 17,204.

The Seventh dataset is BRCA2, in which we compared stage I and stage VI samples regarding BRCA2. This dataset involves 138 samples in total from TCGA, among which 127 are stage I and 11 are stage VI. The number of genes is 17,190.

The Eighth dataset is BRCA3, in which normal samples were compared with samples at stage I in terms of BRCA3. It involves 223 samples in total from TCGA, among which 127 samples are stage I and 96 are normal samples.

### Performance evaluation

The area under the ROC curve (AUC) [22–24] is a statistical method that is employed to assess the discrimination ability of the model. It can be interpreted as a tradeoff between specificity and sensitivity [25]. In this work, we utilize the averaged AUC measured by 5-fold cross-validation run 10 times to assess the performance.

### Experimental results

We first find out the best performance methods in literature including random forest, BP neural network, RBF SVM, linear SVM, Hadamard SVM and RBF MKL, and calculate the optimal parameters and performance of these methods.

**Fig. 3:**
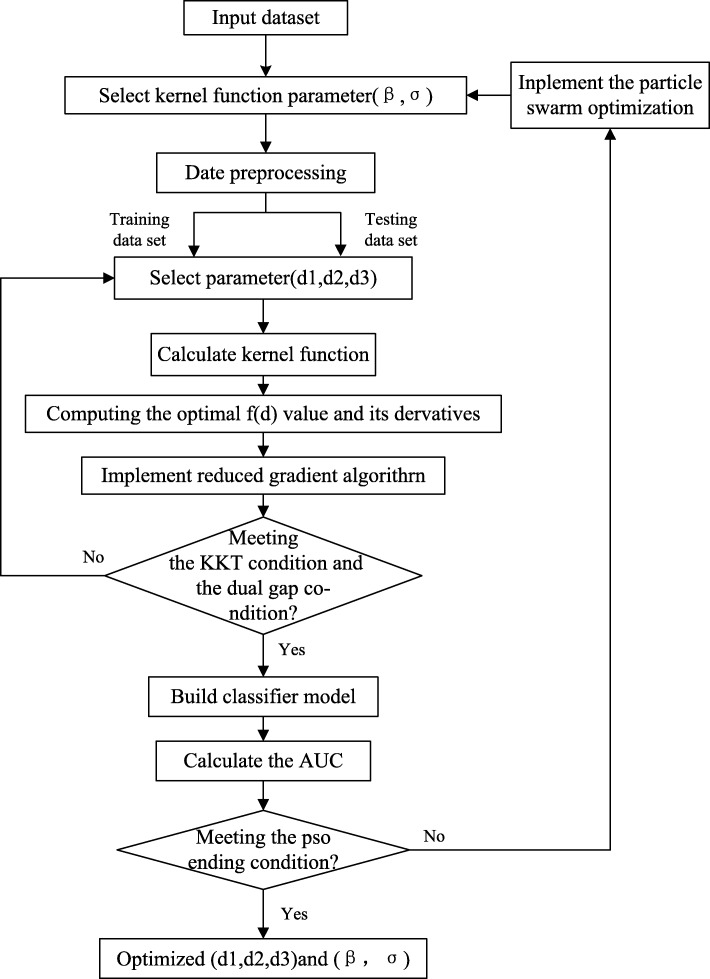
The HMKL flow chart

We propose and improve four schemes. First, Hadamard MKL is a combination of the Hadamard kernel and MKL. Mixed kernels MKL uses the linear, RBF and Hadamard kernels in the MKL framework at the same time. In addition, the number of kernels in the mixed kernels MKL increased to 21 (d = 21). PSO of MKL is used to optimize the kernel function parameters of mixed kernels MKL. Figure 3 shows the HMKL flow chart.

The overall performance of the Hadamard kernels in the experiment is better than that of the linear and RBF kernels. In addition, the gene datasets contain a large number of different genes, which require mixed kernels. MKL has the ability to select an optimal kernel and parameters from a larger set of kernels, reducing the bias due to the kernel selection while allowing for more automated machine learning methods. Therefore, Hadamard MKL uses the Hadamard kernel and achieves better performance than traditional MKL, by using linear, RBF and Hadamard kernels. In order to observe the effect of the increased kernels in MKL, mixed kernels MKL (d = 21) uses a linear kernel, nine RBF kernels and nine Hadamard kernels. Since mixed kernels MKL needs to set the kernel function parameters, HNKL uses PSO to select them.

We show the performance of HMKL, MKL and SVM for the breast cancer evaluation by employing the averaged AUC measured by 5-fold cross-validation run 10 times to assess its performance. Before training the SVM model, we must first specify the kernel function parameters including *σ* of the RBF kernel and *β* of the Hadamard kernel. In general, the choice of the kernel function parameters of the SVM has an impact on the evaluation performance. Firstly, we determine whether the SVM performance is sensitive to the kernel function parameters, and then find the optimal kernel function parameters for the kernel and SVM. Regarding the RBF kernel, we primarily specify the parameter *σ* ∈ {0.01, 0.1, 1, 10, 100, 1000} and conduct 10 times 5-fold cross-validation on the SVM. The results are shown in Table 2, such that the average AUC value is on the left side of the cells, and the corresponding standard deviation is after it. For instance, in the GSE32394 dataset, the SVM performance is extremely sensitive to different values of the parameter σ, while this is not the case in GSE1872.

**Table 2:** Averaged AUC values for determining the optimal σ in the RBF kernel

Datasets	σ = 0.01	σ = 0.1	σ = 1	σ = 10	σ = 100	σ = 1000
GSE32394	0.1589 ± 0.1189	0.1511 ± 0.1511	0.1956 ± 0.1400	0.6667 ± 0.1667	0.9344 ± 0.0456	**0.9367 ± 0.0700**
GSE59993	0.3455 ± 0.0637	0.3606 ± 0.1239	0.4286 ± 0.0433	**0.8287 ± 0.0247**	0.6891 ± 0.0412	0.6988 ± 0.0413
GSE1872	0.2697 ± 0.0917	0.2042 ± 0.0686	0.2068 ± 0.0659	0.2432 ± 0.1053	0.2424 ± 0.1061	**0.2458 ± 0.1027**
GSE76260	0.3823 ± 0.0796	0.4224 ± 0.0464	0.3837 ± 0.0937	0.8270 ± 0.0168	**0.8357 ± 0.0213**	0.8337 ± 0.0485
GSE59246	0.4550 ± 0.0543	0.4442 ± 0.0785	0.7543 ± 0.0462	0.7539 ± 0.0334	0.7553 ± 0.0111	**0.7629 ± 0.0094**
BRCA1	0.2565 ± 0.0776	0.2336 ± 0.1205	0.4720 ± 0.1095	**0.9918 ± 0.0060**	0.9659 ± 0.0303	0.9407 ± 0.0951
BRCA2	0.2316 ± 0.0497	0.2377 ± 0.1074	**0.3709 ± 0.1072**	**1.0000 ± 0.0000**	1.0000 ± 0.0000	1.0000 ± 0.0000
BRCA3	0.3410 ± 0.0424	0.3351 ± 0.0335	0.7377 ± 0.1495	**1.0000 ± 0.0000**	**1.0000 ± 0.0000**	**1.0000 ± 0.0000**

Table 2 illustrates the averaged AUC values of the RBF SVM. We find the best performance RBF kernel function parameter *σ* value for SVM in Table 2. For example, the best *σ* value of the RBF kernel for GSE32394 and GSE1872 is 1000, whereas the best *σ* value for GSE76260 is 100, and the best *σ* value for GSE59993 is 10.

Table 3 illustrates the performance of Hadamard SVM. For example, the best value *β* of the Hadamard kernel for GSE32394 and GSE59246 is − 1, whereas it is 1 for GSE59993 and GSE59246. In the Hadamard kernel, we primarily specify the parameter *β* ∈ {−1, −0.1, −0.01, 0.01, 0.1, 1} and conduct 10 times 5-fold cross-validation on SVM. The results are shown in Table 3, such that the average AUC value is on the left side of the cells, and the corresponding standard deviation is on the right side of the cells. For instance, in the GSE59993 dataset, the performance of SVM is sensitive to different values of the parameter *β*, while the performance of SVM in GSE1872 is not sensitive to different values of the parameter *β* from − 1 to 1.

**Table 3:** Averaged AUC values for determining the optimal β of Hadamard SVM

Datasets	β = −1	β = −0.1	β = − 0.01	β = 0.01	β = 0.1	β = 1
GSE32394	**0.9778 ± 0.0222**	0.9500 ± 0.0278	0.9544 ± 0.0433	0.9444 ± 0.0444	0.9356 ± 0.0356	0.9611 ± 0.0389
GSE59993	0.8063 ± 0.0467	0.6904 ± 0.0809	0.7055 ± 0.0555	0.7137 ± 0.0510	0.7113 ± 0.0294	**0.8661 ± 0.0510**
GSE1872	**1.0000 ± 0.0000**	**1.0000 ± 0.0000**	**1.0000 ± 0.0000**	**1.0000 ± 0.0000**	**1.0000 ± 0.0000**	**1.0000 ± 0.0000**
GSE76260	0.8550 ± 0.0220	0.8346 ± 0.0533	**0.8595 ± 0.0126**	0.8313 ± 0.0389	0.8226 ± 0.0706	0.7673 ± 0.0310
GSE59246	**0.8996 ± 0.0250**	0.8994 ± 0.0143	0.8666 ± 0.0168	0.8564 ± 0.0179	0.8888 ± 0.0227	0.8969 ± 0.0250
BRCA1	0.9726 ± 0.0134	0.9758 ± 0.0089	**0.9953 ± 0.0047**	0.9949 ± 0.0051	0.9750 ± 0.0174	0.9782 ± 0.0161
BRCA2	**1.0000 ± 0.0000**	**1.0000 ± 0.0000**	**1.0000 ± 0.0000**	**1.0000 ± 0.0000**	**1.0000 ± 0.0000**	**1.0000 ± 0.0000**
BRCA3	**1.0000 ± 0.0000**	**1.0000 ± 0.0000**	**1.0000 ± 0.0000**	**1.0000 ± 0.0000**	**1.0000 ± 0.0000**	**1.0000 ± 0.0000**

The averaged AUC values of linear SVM are calculated, and the results are reported in Table 4.

**Table 4:** Averaged AUC values of linear SVM

Datasets	
GSE32394	0.9644 ± 0.0422
GSE59993	0.8371 ± 0.0331
GSE1872	0.3977 ± 0.2008
GSE76260	0.7857 ± 0.0629
GSE59246	0.8896 ± 0.0375
BRCA1	0.9598 ± 0.0317
BRCA2	1.0000 ± 0.0000
BRCA3	0.9997 ± 0.0026

The averaged AUC values of the random forest approach are calculated, and the results are reported in Table 5.

**Table 5:** Averaged AUC values of random forest

Datasets	
GSE32394	0.9644 ± 0.0422
GSE59993	0.8371 ± 0.0331
GSE1872	0.3977 ± 0.2008
GSE76260	0.7857 ± 0.0629
GSE59246	0.8896 ± 0.0375
BRCA1	0.9598 ± 0.0317
BRCA2	1.0000 ± 0.0000
BRCA3	0.9997 ± 0.0026

The averaged AUC values of the decision tree approach are calculated, and the results are reported in Table 6.

**Table 6:** Averaged AUC values of decision tree

Datasets	
GSE32394	0.7589 ± 0.2256
GSE59993	0.8099 ± 0.0740
GSE1872	1.0000 ± 0.0000
GSE76260	0.8313 ± 0.0813
GSE59246	0.8372 ± 0.0497
BRCA1	0.9925 ± 0.0115
BRCA2	0.9997 ± 0.0026
BRCA3	1.0000 ± 0.0000

Table 7 illustrates the averaged AUC values of GA with Rotation Forest.

**Table 7:** Averaged AUC values of GA with Rotation Forest

Datasets	
GSE32394	0.7589 ± 0.2256
GSE59993	0.8099 ± 0.0740
GSE1872	1.0000 ± 0.0000
GSE76260	0.8313 ± 0.0813
GSE59246	0.8372 ± 0.0497
BRCA1	0.9925 ± 0.0115
BRCA2	0.9997 ± 0.0026
BRCA3	1.0000 ± 0.0000

The averaged AUC values of BFA + RF are calculated, and the results are reported in Table 8.

**Table 8:** Averaged AUC values of BFA + RF

Datasets	
GSE32394	0.8000 ± 0.2449
GSE59993	0.8474 ± 0.1381
GSE1872	1.0000 ± 0.0000
GSE76260	0.8167 ± 0.1856
GSE59246	0.7646 ± 0.1304
BRCA1	0.9909 ± 0.2727
BRCA2	1.0000 ± 0.0000
BRCA3	1.0000 ± 0.0000

Table 9 shows the averaged AUC values for all the different methods. For instance, in the GSE32394 breast cancer outcome evaluation, the linear and Hadamard kernels perform better than the RBF kernel in SVM. The Hadamard kernel’s averaged AUC value outperforms that of the RBF kernel, but the Hadamard kernel’s corresponding standard deviation is larger than that of the RBF kernel. The Hadamard kernel MKL outperforms the linear kernel SVM, RBF kernel SVM and Hadamard kernel SVM. Moreover, the mixed kernels MKL outperforms the Hadamard kernel MKL. HMKL outperforms the mixed kernels MKL.

**Table 9:** Averaged AUC values for different methods

Classifier	Decision Tree	Random Forest	GA with Rotation Forest	BFA + RF	SVM	SVM
Kernel					Linear kernel	RBF kernel
GSE32394	0.7589 ± 0.2256	0.8000 ± 0.2449	0.7000 ± 0.3317	0.8000 ± 0.2449	0.9644 ± 0.0422	0.9344 ± 0.0456
GSE59993	0.8099 ± 0.0740	0.7484 ± 0.1438	0.8663 ± 0.0983	0.8474 ± 0.1381	0.8371 ± 0.0331	0.8287 ± 0.0247
GSE1872	1.0000 ± 0.0000	0.9951 ± 0.0178	0.9667 ± 0.1000	1.0000 ± 0.0000	0.3977 ± 0.2008	0.2042 ± 0.0686
GSE76260	0.8313 ± 0.0813	0.7889 ± 0.0441	0.8583 ± 0.0500	0.8167 ± 0.1856	0.7857 ± 0.0629	0.8357 ± 0.0213
GSE59246	0.6455 ± 0.0795	0.8486 ± 0.0349	0.8474 ± 0.1026	0.7646 ± 0.1304	0.8896 ± 0.0375	0.7629 ± 0.0094
BRCA1	0.9925 ± 0.0115	0.9727 ± 0.4166	0.9818 ± 0.3636	0.9909 ± 0.2727	0.9598 ± 0.0317	0.9918 ± 0.0060
BRCA2	0.9997 ± 0.0026	1.0000 ± 0.0000	1.0000 ± 0.0000	1.0000 ± 0.0000	1.0000 ± 0.0000	1.0000 ± 0.0000
BRCA3	1.0000 ± 0.0000	1.0000 ± 0.0000	1.0000 ± 0.0000	1.0000 ± 0.0000	0.9997 ± 0.0026	1.0000 ± 0.0000
Classifier	SVM	MKL(d = 3)	MKL(d = 3)	MKL(d = 3)	MKL(d = 21)	HMKL
Kernel	Hadamard kernel	RBF kernel	Hadamard kernel	Mixed kernels	Mixed kernels	
GSE32394	0.9778 ± 0.0222	0.9422 ± 0.0422	0.9844 ± 0.0511	0.9867 ± 0.6333	0.9899 ± 0.0333	**0.9933 ± 0.0378**
GSE59993	0.8661 ± 0.0510	0.7073 ± 0.0532	0.8973 ± 0.0445	0.8990 ± 0.0336	0.9018 ± 0.0175	**0.9069 ± 0.0178**
GSE1872	1.0000 ± 0.0000	0.2667 ± 0.0894	1.0000 ± 0.0000	1.0000 ± 0.0000	1.0000 ± 0.0000	**1.0000 ± 0.0000**
GSE76260	0.8595 ± 0.0126	0.8302 ± 0.0419	0.8467 ± 0.0313	0.8604 ± 0.0416	0.8633 ± 0.0313	**0.8735 ± 0.0190**
GSE59246	0.8996 ± 0.0250	0.8939 ± 0.0317	0.8991 ± 0.0179	0.9006 ± 0.0292	0.9008 ± 0.0282	**0.9048 ± 0.0047**
BRCA1	0.9953 ± 0.0047	0.9921 ± 0.0061	0.9953 ± 0.0045	0.9957 ± 0.0032	0.9960 ± 0.0026	**0.9967 ± 0.0027**
BRCA2	1.0000 ± 0.0000	1.0000 ± 0.0000	1.0000 ± 0.0000	1.0000 ± 0.0000	1.0000 ± 0.0000	**1.0000 ± 0.0000**
BRCA3	1.0000 ± 0.0000	1.0000 ± 0.0000	1.0000 ± 0.0000	1.0000 ± 0.0000	1.0000 ± 0.0000	**1.0000 ± 0.0000**

We show the performance of HMKL, MKL and SVM for the breast cancer evaluation, such that the parameter values of the developed PSO are set as follows. The cognitive learning factor c1 is set to 1.5, the social learning factor c2 is set to 1.7, the number of particles is 3 and the number of generations is 20. For SVM, we select the optimal parameters and performance of the mixed kernels. In KML, the first part is to utilize only a single type of kernels, which is named single kernel MKL, such as the RBF kernel MKL and Hadamard kernel MKL. The second part is to employ three different types of kernels together, which is named the mixed kernels MKL. d represents the number of kernels in the MKL. When d = 3, the mixed kernels include an RBF kernel, a Hadamard kernel and a linear kernel. When d = 21, the mixed kernels include ten RBF kernels, ten Hadamard kernels and a linear kernel. In HKML, a Hadamard kernel and a linear kernel are utilized.

In the GSE59993 dataset, the Hadamard kernel performs better than the random forest, decision tree, GA with Rotation Forest, BFA + RF, linear kernel SVM and RBF kernel SVM. The Hadamard kernel MKL outperforms the Hadamard kernel SVM. However, the RBF kernel MKL performs worse than the RBF kernel SVM. In addition, the mixed kernels MKL outperforms the single kernel MKL. HMKL outperforms all the other classifiers. In the GSE1872 dataset, the performance of the decision tree, BFA + RF, Hadamard SVM, MKL and HMKL are the best with an AUC of 1. In the GSE76260 dataset, the Hadamard kernel performs better than the random forest, decision tree, GA with Rotation Forest, BFA + RF, RBF and linear kernel in SVM. The Hadamard kernel MKL and RBF kernel MKL outperform the Hadamard kernel SVM and RBF kernel SVM, respectively. In addition, the mixed kernels MKL outperforms the single kernel MKL. HMKL outperforms all the other classifiers. In the GSE59246 dataset, the Hadamard kernel outperforms the GA with Rotation Forest, BFA + RF, decision tree, RBF kernel SVM and linear kernel SVM. The Hadamard kernel MKL outperforms the Hadamard kernel SVM. However, the RBF kernel MKL has a worse performs than the RBF kernel SVM. In addition, the mixed kernels MKL outperforms the single kernel MKL, and HMKL outperforms the mixed kernels MKL. In BRCA1, the Hadamard kernel SVM performs better than the random forest, decision tree, GA with Rotation Forest, BFA + RF, RBF kernel SVM and linear kernel SVM. The Hadamard kernel MKL outperforms the Hadamard kernel SVM. However, the RBF kernel MKL performs worse than the RBF kernel SVM. In addition, the mixed kernels MKL outperforms the single kernel MKL. HMKL outperforms the mixed kernels MKL. In BRCA2 and BRCA3, the performance of the averaged AUC values for different methods is almost the same.

### Analysis and discussion

Based on the previous analysis, we can get the following conclusions:

1. The Hadamard kernel outperforms the RBF and linear kernels for SVM. In the single kernel MKL, the Hadamard kernel outperforms the RBF kernel. In [14], JH calculated the results only when the value of β is positive. On this basis, we find that a negative value of β performs better than a positive one in the Hadamard kernel SVM in GSE32394, GSE59246 (β = − 1) and GSE76260, BRCA1 (β).

2. In the single kernel MKL and SVM, the Hadamard kernel MKL outperforms the Hadamard kernel SVM in all the microarray datasets. It represents that multiple Hadamard kernels outperform a single Hadamard kernel; thus, multiple Hadamard kernels are effective for MKL in the breast cancer microarray datasets.

3. In MKL, the mixed kernels MKL outperforms the single kernel MKL in all the datasets. It represents that multiple heterogeneous kernels are more efficient than multiple single kernels for the breast cancer outcome evaluation. In addition, in heterogeneous kernels MKL, 21 kernels MKL outperforms 3 kernels MKL; thus, more kernels can improve the performance of MKL.

4. The best performance is achieved by HMKL, which surpasses the other methods in terms of performance. It represents that the PSO’s parameter selection is effective for HMKL and can be used to obtain the optimal parameters (*σ*, *β*).

5. Due to the ability of HMKL to optimize the mixed kernel set and its parameters, reducing the bias due to the kernel selection while allowing for more automated machine learning methods, the HMKL performance is better than traditional methods in gene datasets with complex high-dimensional distribution structure. The combination space of mixed kernels (linear, RBF and Hadamard kernels) mappings in HMKL has the ability of feature mapping in each subspace, which ultimately enables the data to be more accurately and reasonably expressed in the new combination space, thus improving the classification performance of HMKL. For different datasets, PSO selects the kernel function in HMKL to improve the classification performance of HMKL.

## Conclusion

In this article, we investigate the effect of the normalization strategy on our proposed HMKL method. It is a valid and effective method for dealing with high dimensional gene expression data when they have positive values. By testing on real-world microarray datasets, HMKL outperforms classical SVM and MKL. In addition, we show that the PSO’s parameter selection is effective for HMKL and can be used to obtain the optimal kernel parameters (*σ*, *β*). For MKL, we show that multiple heterogeneous kernels are more efficient than multiple single kernels. We hope that HMKL can contribute to the wider biological problems as a novel class of methods.

## Data Availability

All the datasets are publicly accessible through The Cancer Genome Atlas and National Center for Biotechnology Information Gene Expression Omnibus, where the accession number are GSE32394, GSE59993, GSE1872, GSE76260 and GSE59246.

## Abbreviations

AUC: Area under curve
SVM: Support vector machines
PSO: Particle swarm optimization
MKL: Multiple kernel learning
HMKL: Heterogeneous multiple kernel learning
